# Metagenome data of bacterial diversity in pear (*Pyrus communis* L.) rhizospheres associated with *Phytophthora* infection and amino acid treatment

**DOI:** 10.1016/j.dib.2019.104396

**Published:** 2019-08-24

**Authors:** Antonios Zambounis, Maslin Osathanunkul, Panagiotis Madesis

**Affiliations:** a Institute of Plant Breeding and Genetic Resources, Department of Deciduous Fruit Trees, HAO, ‘Demeter’, Naoussa, Greece; b Department of Biology, Faculty of Science, Chiang Mai University, Chiang Mai, 50200, Thailand; c Center of Excellence in Bioresources for Agriculture, Industry and Medicine Chiang Mai University, Chiang Mai, 50200, Thailand; d Institute of Applied Biosciences (INAB), CERTH, 6th Km Charilaou-Thermi Road, 57001, Thessaloniki, Greece

**Keywords:** Deep amplicon sequencing, Metagenomics, 16S bacteriome communities, *Phytophthora*, Oomycetes

## Abstract

The bacterial diversity in rhizosphere soil of pear trees (*Pyrus communis* L. cv. *Krystalli*) from an orchard at Thessaly region of Greece was characterized employing amplicon-based metagenomics analysis. Pathogenic filamentous oomycetes of the genus *Phytophthora* comprises more than 150 recognized species and cause highly destructive soil-borne diseases in deciduous trees crops worldwide. Moreover, the treatment of soil microbiota with amino acids is an alternative strategy to achieve desirable effects even against phytopathogenic oomycetes. In our study, samples from rhizosphere soil were collected either from naturally *Phytophthora*-infected trees, from completely asymptomatic ones, or from trees as above subjected also to treatments with amino acids (Amino16®) under different fertilization regimes. The interactions of bacterial communities with plant pathogenic oomycetes are crucial to determine the course of infection and the pathogenicity encompassing various functional contexts like biofilm formation. Thus, for deciphering the structure and diversity of these soil bacterial communities, we applied a 16S rRNA Illumina sequencing approach targeting the V3-V4 gene region. After quality check 478,479 sequences were obtained in the dataset comprising a total read length of 192,291,625 base pairs. *Proteobacteria* were the dominant phylum (46.1%) followed by *Acidobacteria* (13.2%) and *Actinobacteria* (12.4%). Different distributions of phyla were observed among our samples which is indicative of various alterations of soil bacterial communities in rhizosphere. The metagenome data from this survey are available at NCBI Sequence Read Archive (SRA) database and Biosample under accession number PRJNA542725.

**Table:** Specifications Table

Subject area	Phytopathology
More specific subject area	Microbiome
Type of data	Table, Text Files, Figures, 16S rRNA sequences and analysis
How data was acquired	NGS sequencing on Illumina HiSeq platform
Data format	Raw and analyzed.
Experimental factors	Bacterial genomic DNA from collected rhizospheres of a pear orchard was extracted and used as template to amplify the V3-V4 region of the 16S rRNA gene
Experimental features	Comparison of soil bacterial communities in rhizosphere of pear trees with absence or presence of visible *Phytophthora* rot symptoms and after amino acids applications
Data source location	The samples were collected from rhizosphere soil in a experimental pear orchard in Tyrnavos, at Thessaly region, Greece (39° 68′ 28.69″ N, 22° 35′ 36.46″ E)
Data accessibility	Data is within this article and all sequences generated in this study are submitted to NCBI SRA under the accession numbers SRR9050902 up to SRR9050907 being available in the NCBI BioSample Submission Portal as Bioproject PRJNA542725 (https://www.ncbi.nlm.nih.gov/bioproject/PRJNA542725)
Related research article	J.G. Caporaso, C.L. Lauber, W.A. Walters, D. Berg-Lyons, C.A. Lozupone, P.J. Turnbaugh, N. Fierer, RL, Knight Global patterns of 16S rRNA diversity at a depth of millions of sequences per sample, PNAS 108 (2011) 4516-4522

**Table:** 

**Value of the data** • These metagenome data provide valuable information about the diversity and structure of rhizosphere bacterial communities associated with naturally *Phytophthora*-infected pear trees, highlighting simultaneously the effects of treatments with amino acids in the alterations of bacterial communities. • The data enhance our understanding on dominant microbial inhabitants of pears rhizospheres that may further be exploited for growing crops under various biotic conditions and fertility soil profiles. • Profiling of bacterial communities using Illumina technology provides a cost effective and efficient, in depth, sequencing, thus offering a useful approach for the study and comparison of bacterial communities across pear orchards. • This project expands our knowledge on bacterial diversity in the rhizospheres of *Phytophthora*-infected pear trees indicating the putative role of plant growth promoting bacteria (PGPB) in pathogenicity of soil-borne diseases. • This culture-independent 16S rRNA amplicon sequencing revealed a relative highly dominance of Actinobacteria in the rhizosphere soil of *Phytophthora*-infected pear trees which might indicate their important role towards novel bioprospecting applications.

## 1 Data

**Fig. 1:**
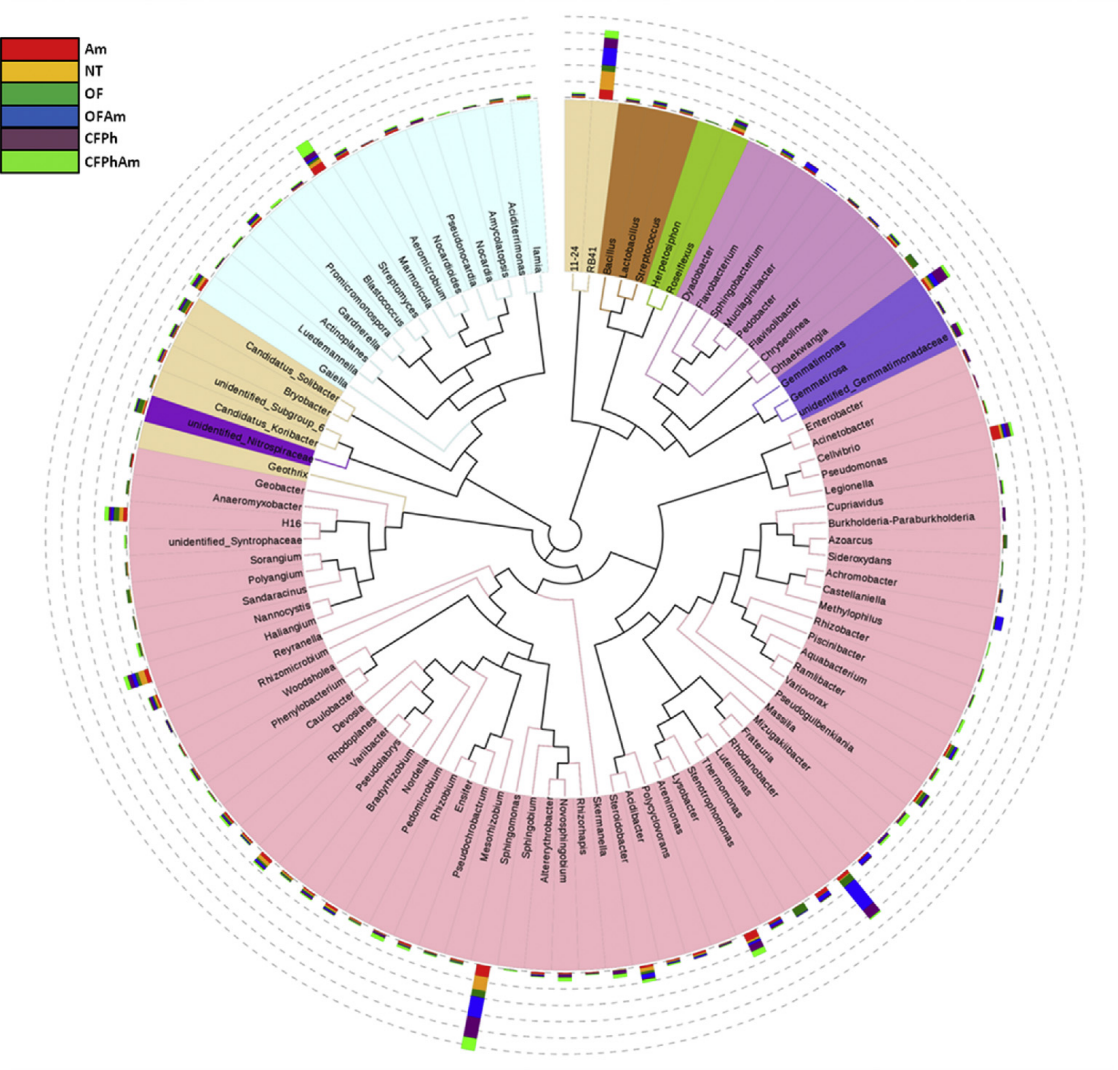
An evolutionary tree in genus level across all six samples of this study. Different colors of the branches represent different phyla. Relative abundance of each genus in each sample was displayed outside the circle and different colors represent different samples.

**Fig. 2:**
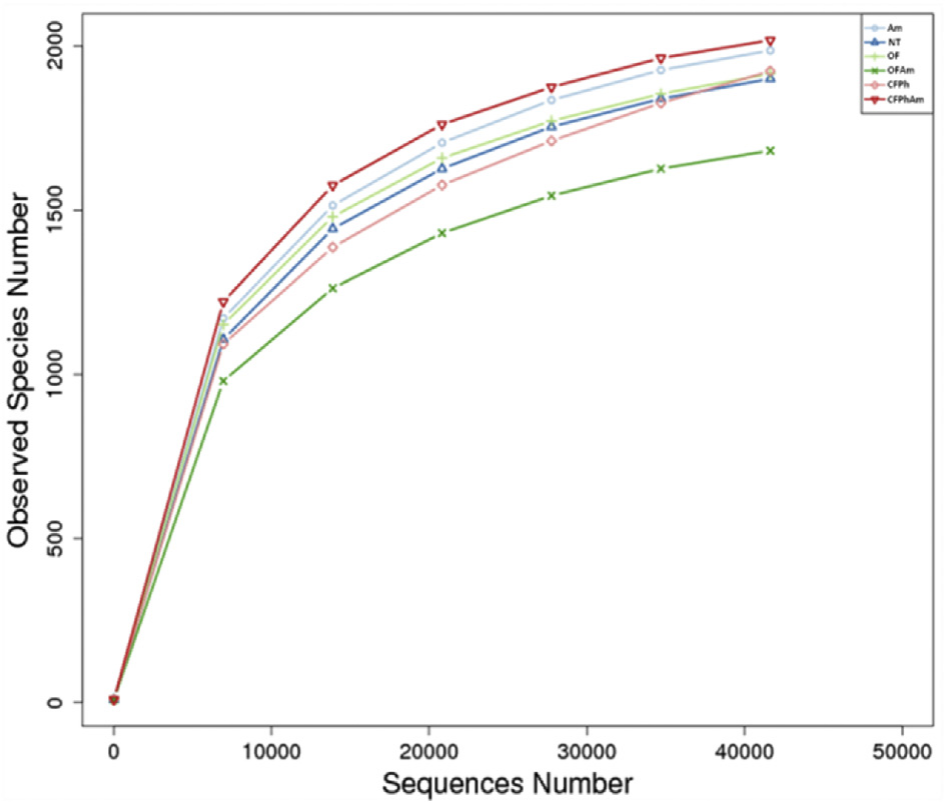
Rarefaction curves with each curve represent a sample. The sequences number is on the X-axis and the observed OTUs number is on the Y-axis. For the rank abundance curves, each curve represents a single sample, plotted by OTU relative abundance on the Y-axis and the OTU abundance rank on the X-axis.

**Fig. 3:**
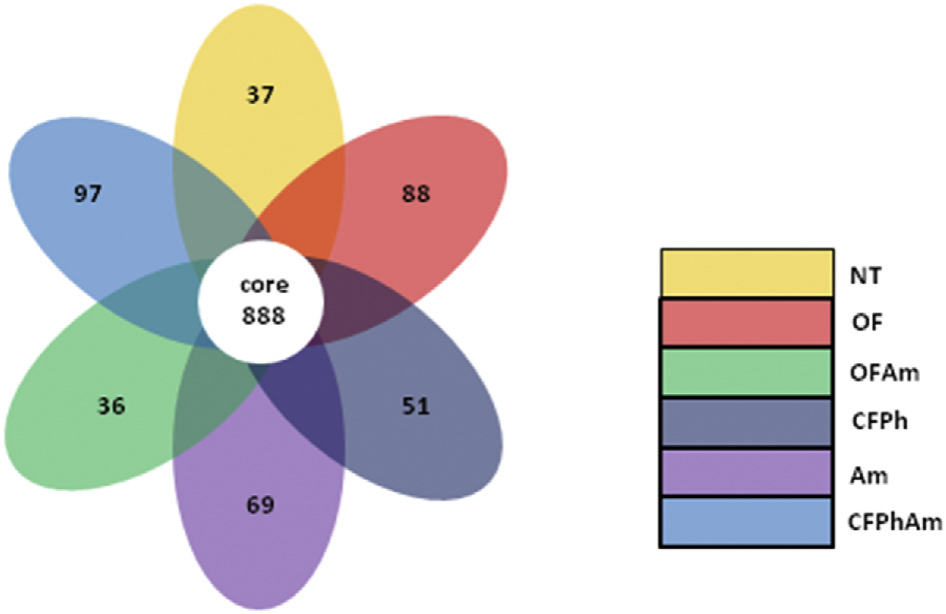
Flower diagram based on OTUs. Each petal represents for each sample. The core number in the center is for the number of OTUs present in all samples, while number in the petal is for the unique OTUs in each sample.

**Fig. 4:**
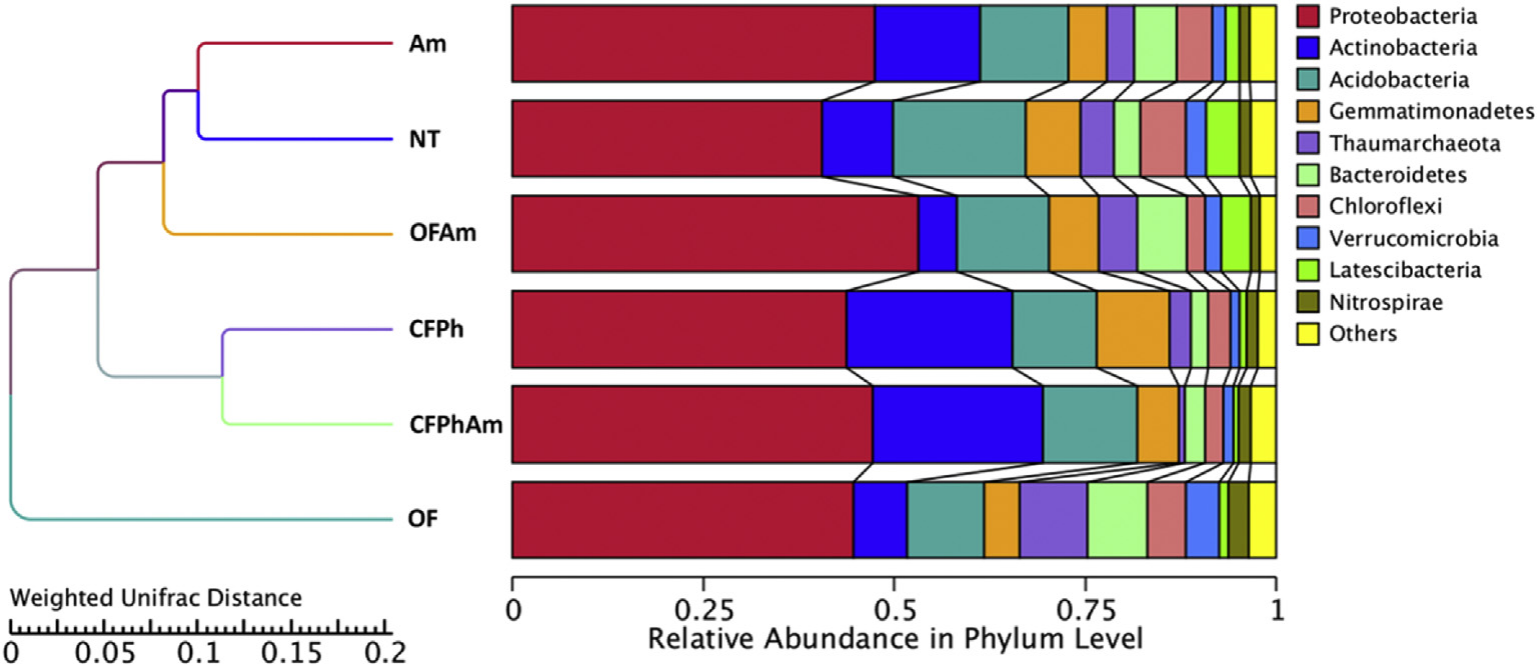
UPGMA cluster tree based on weighted unifrac distance along with species relative abundance and distribution in phylum level.

**Fig. 5:**
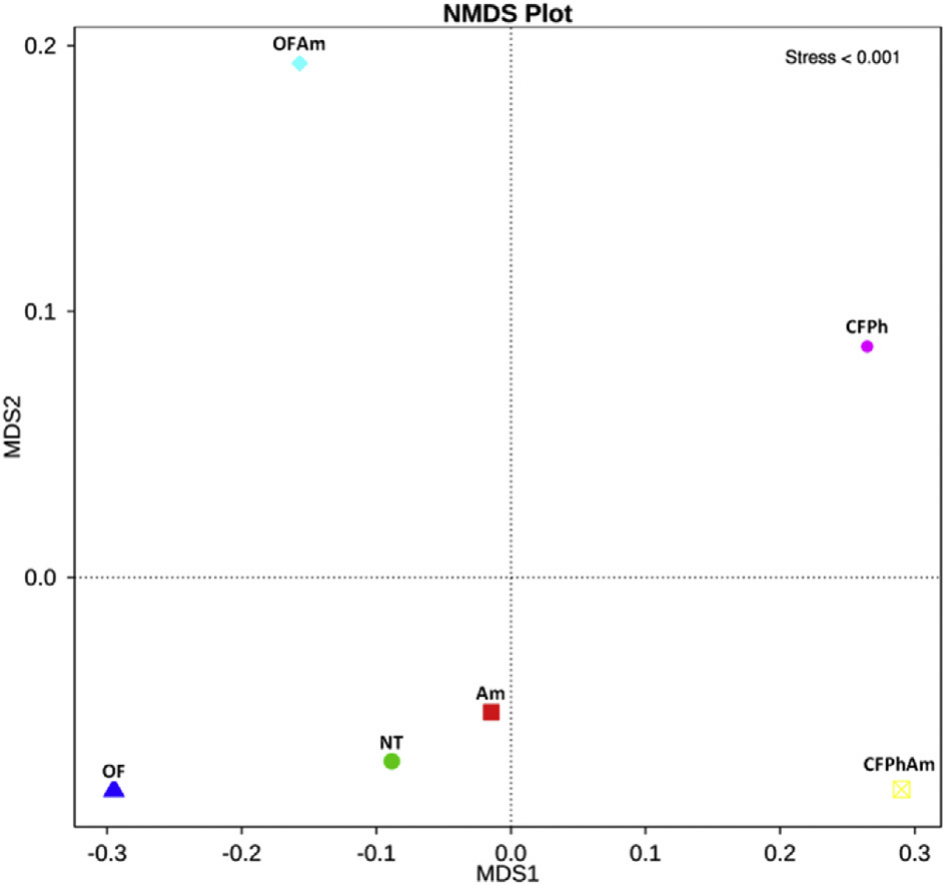
NMDS visualization with distance between data points reflects the extent of variation.

Culture-independent technologies such as high throughput molecular techniques and next generation sequencing (NGS) have accelerated our ability to analyze microbial community composition in various agricultural environments [1]. In this study, we investigated the alterations of soil bacterial communities in rhizosphere of pear trees which were chosen for sampling based on absence or presence of visible *Phytophthora* rot symptoms under naturally infections and after amino acids applications under different fertilization regimes (conventional or organic). This metagenome methodology will further allow deciphering the interactions of rhizosphere bacteriome with oomycete pathogens. In this project, 16S rRNA Illumina sequencing was performed revealing the alterations of bacterial communities from phylum to family in composition and specificity of the soil rhizopheric microbiota. An evolutionary tree (Fig. 1) displays the relative abundance of each genus for all samples based on the phylogenetic relationships between all Operational Taxonomic Units (OTUs) representative sequences. Fig. 2 and Table 1 provide the species diversity by rarefaction curves and the overview of the bacterial community structure. Fig. 3 shows a flower diagram based on OTUs distribution for each sample, while an UPGMA cluster tree which was based on Weighted Unifrac distances, species relative abundance and distribution in phylum level is shown in Fig. 4. The visualization of a Non-Metric Multi-Dimensional Scaling (NMDS) with distance between data points to reflect the extent of variation is depicted in Fig. 5. The data are useful for understanding the bacterial diversity associated with *Phytophthora* decline of pear trees, and how fertilization with amino acids may influence the dynamics of bacterial communities.

**Table 1:** Details about the Illumina sequencing metagenome analysis of soil bacterial communities in rhizospheres of pear trees.

Sample_name	Total_reads (PE)	Combined_reads	Combined_base(bp)	Avg_len(bp)	observed_species	Shannon	simpson	Chao 1 richness
Am	136,166	118,967	49,753,064	418	1986	8.957	0.995	2.134.642
CFPhAm	120,385	106,746	44,617,003	418	2017	9.196	0.996	2.137.587
CFPh	122,227	107,241	44,772,315	417	1923	8.828	0.994	2.931.418
NT	138,077	122,025	51,022,359	418	1900	8.648	0.992	2.077.066
OFAm	128,772	112,541	47,257,703	420	1681	8.018	0.984	1.851.547
OF	131,449	115,483	48,360,587	419	1915	8.721	0.990	2.082.625

## 2 Experimental design, materials and methods

### 2.1 Samples collection

This study was conducted in one of the main pear (*Pyrus communis* L. cv. *Krystalli*) cultivation area of Greece located in Tyrnavos, at Thessaly region (39° 68′ 28.69″ N, 22° 35′ 36.46″ E). Soil samples were collected in April of 2018. These samples were taken systematically using hand gloves from a depth of 30 cm around the rhizosphere of three years-old pear trees at one sampling location (its soil texture type was loam). Soil was gently removed from roots and retained for DNA extraction. The soil samples were transferred into sterilized polythene bags and stored in ice box for transportation to the laboratory, where they stored at −80 °C until DNA extraction. Samples were collected either from pear trees which were completely asymptomatic with absence of any visible *Phytophthora* rot symptom, or from naturally *Phytophthora-*infected pear trees with visible lateral root discolouration and lesions extended up to crown. The healthy pear trees were constantly received either conventional N-fertilization (CT) or organic fertilization (OF), whereas the naturally infected trees were previously taken conventional fertilization (CFPh). Samples from rhizosphere soil were also received from trees in the same sampling orchard, as above, which were previously received a soil drenching with an amino acids solution (0.5% Amino16®) around their trunk. These treatments were performed twice at equal doses every 15 days for the last one month before the sampling collection. Thus, applications with amino acids were applied either in healthy pear trees under conventional fertilization regime (Am), under organic fertilization regime (OFAm), or in naturally *Phytophthora*-infected pear trees (CFPhAm). Three distinct replicates per condition (treatment) were performed and the soil samples from each condition were pooled to create one sample, six in total in their number.

### 2.2 DNA extraction

Metagenomic DNA was extracted from 0.25 g of soil samples using the NucleoSpin® Soil Kit (Macherey-Nagel, Germany), following the manufacturer's instructions. DNA was quantified in a NanoDrop spectrophotometer. DNA quantity and purity were also verified by electrophoresis in 1.2% agarose gels and concentration of samples adjusted equally at 50 ng/ul.

### 2.3 Libraries preparation and amplicons generation

The 16S rRNA genes spanning the V3-V4 region were amplified by employing the barcoded primer pair 341F (CCTAYGGGRBGCASCAG)/806R (GGACTACNNGGGTATCTAAT) [2]. All PCR reactions were carried out with Phusion® High-Fidelity PCR Master Mix (New England Biolabs). Illumina sequencing libraries were generated using NEB Next® Ultra DNA Library Pre Kit by adding index codes. The quality of libraries was assessed on the Qubit® 2.0 Fluorometer (Thermo Scientific) and Agilent Bioanalyzer 2100 system. The sequencing of amplicons was performed on an Illumina platform and 250 bp paired-end reads were generated which were subsequently merged using FLASH v.1.2.7 [3]. A quality filtering on the raw tags was performed in order to obtain the high-quality clean tags according to the QIIME v.1.7.0 [4] based on the quality control process. The tags were compared with the reference Gold database using the UCHIME algorithm [5] in order to remove chimera sequences and obtain the effective tags (Table 1). Their average length was 418 bp and the observed species for each sample were ranging from 2,017 (CFPhAm) to 1,681 (OFAm).

### 2.4 OTU clustering, species annotation, taxon relative abundance and phylogenetic construction

In order to analyze the species diversity in each sample, all effective tags were grouped by 97% DNA sequence similarity into OTUs, using the Uparse software v.7.0.1001 [6]. Mothur software package was employed for species annotation at each taxonomic rank against the SSU rRNA database of SILVA Database [7]. The top 10 taxons in the different taxonomic ranks were selected to form the distribution histogram of relative abundance. Subsequently, the MUSCLE software v.3.8.31 [8] was employed to create the phylogenetic relationships between all OTUs representative sequences. The tree graph of species annotation for each sample was constructed by GraPhlAn software [9]. Finally, the top 100 genera were selected and an evolutionary tree displaying the relative abundance of each genus was drawn using all the aligned represent sequences (Fig. 1).

### 2.5 Diversity analyses and indices

Alpha diversity was calculated with QIIME pipeline in order to analyze the complexity of species diversity (Table 1). Rarefaction and rank abundance curves (Fig. 2) were used to indicate the biodiversity of the samples and for visualization of species richness and evenness [10]. According to OTUs clustering, we analyzed both the common and unique information for all samples, and a Flower diagram was generated (Fig. 3). Thus, among the 1,266 meaningful and clustered OTUs, almost 31% of them were unique across all the six samples. In samples CFPh and CFPhAm which were both corresponding to the rhizosphere of the naturally *Phytophthora-*infected trees, the total number of unique OTUs was 148, almost 39% of the total number of unique OTUs across samples.

Beta diversity analysis was employed to evaluate the complexity differences between our samples in terms of species complexity. An unweighted pair-sample UPGMA clustering was performed as a type of hierarchical clustering method to interpret the distance matrix using average linkage. Weighted unifrac distance matrix was calculated before being used for UPGMA cluster analysis (Fig. 4). All the metastats were calculated in R software v.2.15.3. These results revealed that samples CFPh and CFPhAm were closely clustered implying that such naturally infections promote a rather distinct bacteriome profile. Besides, the phylum of *Actinobacteria* was relatively abundant and mostly distributed across these two samples. In contrary, the distribution of phylum *Thaumarchaeota* was rather decreased in the above two samples compared with its relative abundance among the other samples. *Proteobaceria* were found to be more abundant across all the amino acids applications (Am, OFAm, CFPhAm). Finally, a Non-Metric Multi-Dimensional Scaling (NMDS) plot was performed (Fig. 5) which was based on a nonlinear ranking model in order to provide an explanation of the nonlinear structure of our dataset [11].

Hence, additional studies are required in order to enlarge our knowledge about the soil bacterial communities in rhizosphere of deciduous trees associated with naturally infected trees from oomycetes of the genus *Phytophthora* and after various soil treatments under different fertilization regimes. Such approaches will allow us to decipher the interactions between rhizosphere bacteriome and plant-pathogenic oomycetes.
